# Ethnic inequities in mathematical models for respiratory infectious disease: A systematic review

**DOI:** 10.1017/S0950268826102118

**Published:** 2026-10-15

**Authors:** Zoe Kumbaroff, Emily P. Harvey, Jesse Whitehead, Dion R.J. O’Neale

**Affiliations:** 1 Te Ngira Institute for Population Research, University of Waikato, New Zealand; 2 Te Pūnaha Matatini, New Zealand; 3 The New Zealand Institute for Public Health and Forensic Science; 4 University of Auckland, New Zealand; 5 Nicholson Consulting, New Zealand

**Keywords:** Ethnicity, Health inequities, Mathematical epidemiology, Modelling and simulation, Disease spread, Compartmental models, Agent based models, Public health, Population demographics, Epidemic burden

## Abstract

COVID-19 was the most recent pandemic to highlight profound ethnic inequities. However, most mathematical epidemiological models do not adequately represent these inequities, limiting the potential for such models to inform equitable public health responses and interventions. This systematic review identifies mathematical models of infectious respiratory disease transmission that report results by ethnic group and surveys the different mathematical modelling approaches used to attempt to capture ethnicity-related outcomes. Only 28 studies met the inclusion criteria, which is rare relative to the volume of infectious disease modelling literature. The most common model frameworks were compartmental models (11 studies), individual-based models (12 studies), and metapopulation models (5 studies). Across these studies, ethnic heterogeneities were represented directly by incorporating some combination of ethnic group population demographics, ethnicity-specific interaction patterns, and ethnicity-specific disease parameters, or indirectly through spatial differences in interaction patterns or disease transmission. Consideration of ethnicity in mathematical models of disease transmission is in its infancy, with only three of the studies published before 2020, but it has the potential to provide improved evidence for public health action that reduces the epidemic burden and inequities across ethnic groups.

## Introduction

Mathematical models of infectious disease transmission grew in frequency of use and in complexity during the COVID-19 pandemic as they proved to be valuable tools for scenario modelling to evaluate potential public health interventions and inform decision-making [1]. However, the design choices and implementation of some mathematical models have been criticized for failing to correctly embed epidemiological principles related to the distribution and determinants of disease spread [2–6] and for failing to discuss the validity of model findings [7].

In general, the burden of any infectious disease is not distributed evenly across population groups. Globally, historically marginalized ethnic and racial groups experience a greater infectious respiratory disease burden than other population groups. In Aotearoa New Zealand (NZ), Māori and Pacific peoples experience higher rates of hospitalization for severe acute respiratory infection [8–10] and African American/Black, Latin American, and Indigenous populations in North America experience higher COVID-19, influenza, and RSV burden [11–14].

These unfair and avoidable differential health outcomes, or ‘health inequities’, can be challenging to study because they are the result of many overlapping factors including housing and working conditions, environmental factors, health care access barriers, and social, political, and geographical factors, which interact, compound, and feedback on each other [15–17]. Mathematical transmission models provide a powerful tool to investigate the effect of different factors on infectious disease dynamics and to estimate the impact of different interventions or policies on the infectious disease burden in a population [18]. By building transmission models that include the key drivers of ethnic differences in the burden of disease and stratify model results by ethnicity, it is possible to develop more equitable public health strategies to manage respiratory infectious disease [6].

Considering ethnicity in acute respiratory illness mathematical models is difficult and must be done carefully and with a nuanced understanding of real-world context. For example, some ethnic groups have experienced historical and ongoing marginalization that has led to increased barriers to accessing health services and other social determinants necessary for good health. These structural factors likely explain many of the differential infectious disease rates and – if not explicitly represented by mechanisms or processes within a model – should at least be considered and discussed as part of mathematical modelling studies [2]. Furthermore, even appropriately quantifying ethnicity within a population can be a challenge for mathematical modelling, and there is often a lack of suitable data to build and calibrate ethnicity-specific mathematical models: case data that are available may not have reliable ethnicity information or may suffer from systemic biases in reporting that correlate with ethnicity; individuals can have multiple ethnicities; information needed to quantify the factors that contribute to the observed inequities in infection rates such as differences in contact patterns may not be available; and even when data related to ethnic differences in respiratory infectious disease are available, the interaction of different factors that might drive differences in infection rates can be difficult to resolve into leading order and secondary effects [3, 5].

To date, we are not aware of any systematic reviews on representations of ethnicity in mathematical infectious disease transmission models. Therefore, the aim of this systematic review is to identify and compare mathematical transmission modelling studies which report results by ethnicity. We restrict this review to mechanistic mathematical models of respiratory disease transmission that can be used for scenario modelling, such as investigating the impact of different policies or interventions, or unknowns such as novel diseases. We exclude statistical and forecasting models, which are focused on forecasting short-term trajectories, nowcasting, or estimating risk factors.

In the studies identified in this systematic review, we investigate how the models represent ethnicity, what additional factors are incorporated, the data sources used, and the potential sources of bias. We identify key gaps and provide recommendations for better practice to inform equity-focused infectious respiratory disease modelling.

## Methods

This study followed the Preferred Reporting Items for Systematic Reviews and Meta-Analyses (PRISMA). The review protocol, including the search method, is published on PROSPERO (number CRD420251063754). No ethical approval was required for this research.

Scopus, ProQuest, EBSCOhost, Wiley, and Cochrane databases were searched on 2 June 2025 (see Supplementary File 1 for the full keyword search for each database). No date filters were applied. Studies modelling HIV and other sexually transmitted and blood-borne pathogens were excluded. Results were imported into EndNote and deduplicated. One author screened titles to identify potentially relevant studies, and then screened abstracts and full texts to determine whether studies were to be excluded or included. A second author used full text to spot-check 10% of the excluded studies and to check all included studies and the data extraction results tables. Coauthors discussed any conflicts regarding included and excluded studies.

We included studies that used a mathematical model to simulate infectious disease dynamics for respiratory pathogens that are primarily transmitted from person to person and that reported model results by ethnic group. We excluded studies that compare populations in different countries or geographic areas without discussion of ethnicity, were not published in English (three studies), or had no full text (seven studies) available.

In this review, we differentiate between three common model frameworks – compartmental, metapopulation, and individual-based models:

- ***Compartmental models*** are the most well-known model type. These models partition the population into groups, or compartments, according to infection status and other variables such as age, location, vaccination status, or ethnicity. Each group within the model can be treated as a homogeneous compartment with its own set of parameters that govern the rates of transition to other compartments and its influence, for example, the spread of disease, on other compartments.
- ***Metapopulation models*** are an extension of compartmental models that allow for multiple populations, each with their own compartmental model, to be linked through contact rates or interaction patterns. A specific type of metapopulation model, commonly used in COVID-19 pandemic modelling, uses populations which are grouped by geographic location (like a state, suburb, or region) that each have their own compartmental model but with additional parameters that govern transmission between each population. The multi-scale structure of metapopulation models enables them to incorporate more detailed population information, for example, spatial heterogeneities, which often includes differences in ethnic proportions.
- ***Individual-based models*** (IBMs) model disease transmission and progression at the level of individuals in the population. This means that IBMs can incorporate population heterogeneities more flexibly by setting individual-level characteristics and interaction patterns. IBMs can also include detailed processes for disease transmission and progression that can depend on the attributes of individuals, their neighbours, and their environment. Limitations of IBMs include that they are computationally demanding to design and run and are difficult to calibrate and validate. They require detailed data to appropriately capture population heterogeneities and the implications for the spread of disease [18–20].

Although ethnicity is a self-determined variable to describe cultural affiliation in most governmental data in New Zealand [21], in the USA, both race and ethnicity are used, with ethnicity used only to differentiate between ‘Hispanic or Latino’ and ‘not Hispanic or Latino’ [22]. For consistency, the US definition of race/ethnicity is referred to only as ‘ethnicity’ in this work.

Information was extracted from the eligible papers related to model design, model purpose, level of demographic granularity, parameters that varied by ethnicity, representation of contact/interaction patterns, data sources, and bias, and recorded in Microsoft Excel.

## Results

**Figure 1.**
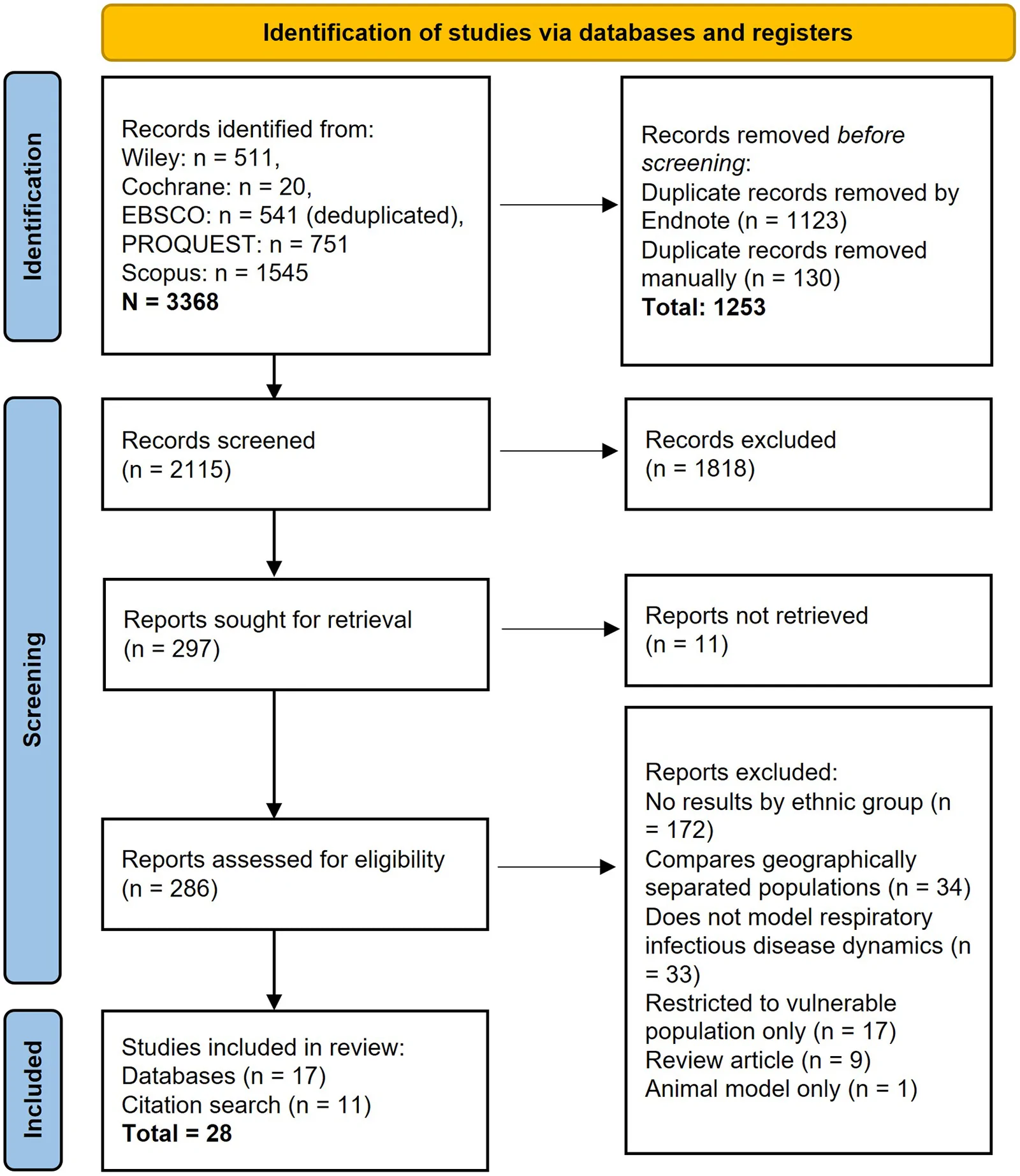
PRISMA flow diagram for systematic reviews. Figure 1. long description.

Figure 1 shows the flow diagram for this systematic review. There were 28 papers that met the inclusion criteria. Seventeen were identified from databases and registers in the initial search, and 11 more were identified from snowball sampling of reference lists and ‘cited-by’ tools on Google Scholar.

Table 1 summarizes the key features of the 28 included studies. Twenty-four studies were peer-reviewed journal articles, two were theses (one Master’s, one PhD) [23, 24], and two were preprints (not peer-reviewed) [31, 51]. Most studies modelled USA populations (22 studies), with other papers modelling New Zealand (2), Canada (1), Australia (1), United Kingdom (1), and hypothetical (1) populations. Eleven papers used a compartmental model, 12 used an IBM, and 5 used a metapopulation model. We found that all 12 IBMs and 4 out of 5 metapopulations used stochastic transmission processes. In contrast, only 2 out of 11 compartmental models simulated stochastic transmission processes, with the majority being deterministic models.

**Table 1.** Data extraction summary for studies included in this systematic review Table 1. long description.

Author	Geographical setting	Model type	Disease and immunity states	Deterministic vs. stochastic	Pathogen	Model purpose
Arigbede [23]	USA	IBM	6	Stochastic	Influenza and COVID–19	Predict emergency department visits and health service utilization during outbreaks
Barnes [24]	USA	CM	3	Deterministic	COVID–19	Examine relationships between the social determinants of health and COVID–19 infection rates
Bilal et al. [25]	Hillsborough, Florida, USA	IBM	14	Stochastic	COVID–19	Demonstrate a city-scale disease transmission model for pandemic simulation
Chang et al. [26]	USA (10 largest metropolitan areas)	MM	4	Stochastic	COVID–19	Investigate the relationship between mobility, COVID–19 transmission, and inequities
Chen et al. [27]	USA (9 large metropolitan areas)	MM	4	Stochastic	COVID–19	Develop vaccination strategies to improve both equity and utility
Datta et al. [28]	NZ	CM	14	Deterministic	COVID–19	Investigate vaccination rate counterfactual scenarios
Elderd et al. [29]	Southwest USA, Mexico, New England, England, and Mauritius (13 locations of smallpox epidemics)	CM	4	Deterministic	Smallpox	Look for evidence of differences in R0 in different populations from historic smallpox outbreak data
Engebretsen et al. [30]	Hypothetical	CM	3	Stochastic	Agnostic, inspired by COVID–19	Demonstrate the limitations of regression analysis for estimating individual-level risk in heterogeneous connected populations and highlight the importance of community-level risk considerations for infectious disease spread
Fleurantin et al. [31	USA (9 major states)	CM	12	Deterministic	COVID–19	Develop a model for SARS-CoV–2 transmission that can be used for pandemic planning and evaluating pandemic response
Gyorgy et al. [32]	Chicago, IL, USA	MM	4	Stochastic	COVID–19	Understand the relationship between neighbourhood segregation and transmission dynamics
Hou et al. [33]	Wisconsin, USA	MM	4	Stochastic	COVID–19	Investigate mobility and demographic influence on disease transmission
Kadelka et al. [34]	USA	CM	20	Deterministic	COVID–19	Assess the impact of different assumptions around ethnic and occupation-based homophily on the modelling results related to COVID–19 vaccine allocation
Klotz et al. [35]	Quebec, Canada	CM	5	Deterministic	Tuberculosis	Forecast tuberculosis incidence over the long term for three different population groups
Ma et al. [36]	New York City and Long Island, NY, USA	CM	4	Deterministic	COVID–19	Estimate the different contact rates by ethnic group and implications for vaccination strategies under assumptions of different levels of assortativity in ethnic group mixing
Owusu-Dampare and Bouchnita [37]	El Paso, TX, USA	CM	9	Stochastic	COVID–19	Evaluate the impact of different vaccination strategies on COVID–19 disparities for Hispanic populations
Patel et al. [38]	North Carolina State, USA	IBM	7	Stochastic	COVID–19	Evaluate the impact of vaccination and other interventions on infection, hospitalization, and mortality rates
Quera-Bofarull et al. [40]	London, UK	IBM	9	Stochastic	COVID–19	Demonstrate a city-scale disease transmission model for pandemic simulation
Rosenstrom et al. [41]	North Carolina State, USA	IBM	7	Stochastic	COVID–19	Evaluate the impact of different vaccination strategies on disparities
Sivakumaar et al. [42]	USA (3 states)	IBM	4	Stochastic	COVID–19	Evaluate the impact of different non-pharmaceutical interventions on COVID–19 inequities
Stafford et al. [43]	Oregon, USA	CM	18	Deterministic	COVID–19	Evaluate different vaccination strategies under different mixing assumptions and for different objectives (minimizing deaths vs. minimizing years of life lost)
Stafford et al. [44]	USA (representative population)	IBM	7	Stochastic	Influenza	Evaluate the impact of different interventions on influenza inequities
Starr and Kain [45]	USA (4 small counties)	IBM	8	Stochastic	COVID–19	Investigate how social determinants of health drive inequitable COVID–19 outcomes and how local interventions can be designed to improve health equity
Thakur et al. [46]	Virginia, USA	IBM	5	Stochastic	Measles	Evaluate the impact of different vaccination scenarios on disparities
Thomas et al. [47]	San Francisco, CA, USA	IBM	5	Stochastic	COVID–19	Investigate the role of social cohesion and spread (specifically cliques in contact networks, and ’core number’) in early stages of an epidemic
Vino et al. [48]	Australia (13 shire counties and 2 local government areas)	CM	4	Deterministic	Influenza-like-illness	Demonstrate methods to collect household contact data, produce group-specific household contact matrices, and use these in a disease transmission model
Wang et al. [49]	Greater Boston, Massachusetts, USA	MM	3	Deterministic	COVID–19	Investigate vaccination intentions, racial disparities, and COVID–19 spread
Williams et al. [50]	USA (8 counties)	IBM	6	Stochastic	Influenza	Estimate the effect of influenza vaccination disparities
Zhang et al. [51]	NZ (3 health districts)	IBM	3	Stochastic	Measles	Demonstrate the use of an IBM calibrated using machine learning techniques for measles outbreak simulation and intervention evaluation

CM, compartmental model; IBM, individual-based model; MM, metapopulation model.

The number of disease states varied widely across models depending on the purpose of the study and the disease being studied. The compartmental models used 3–20 states, the metapopulation models used 3–4 states, and the IBMs used 3–14 disease states. Models included more disease states when more detail was needed on disease progression, such as hospitalizations and deaths, or on different vaccination states.

**Figure 2.**
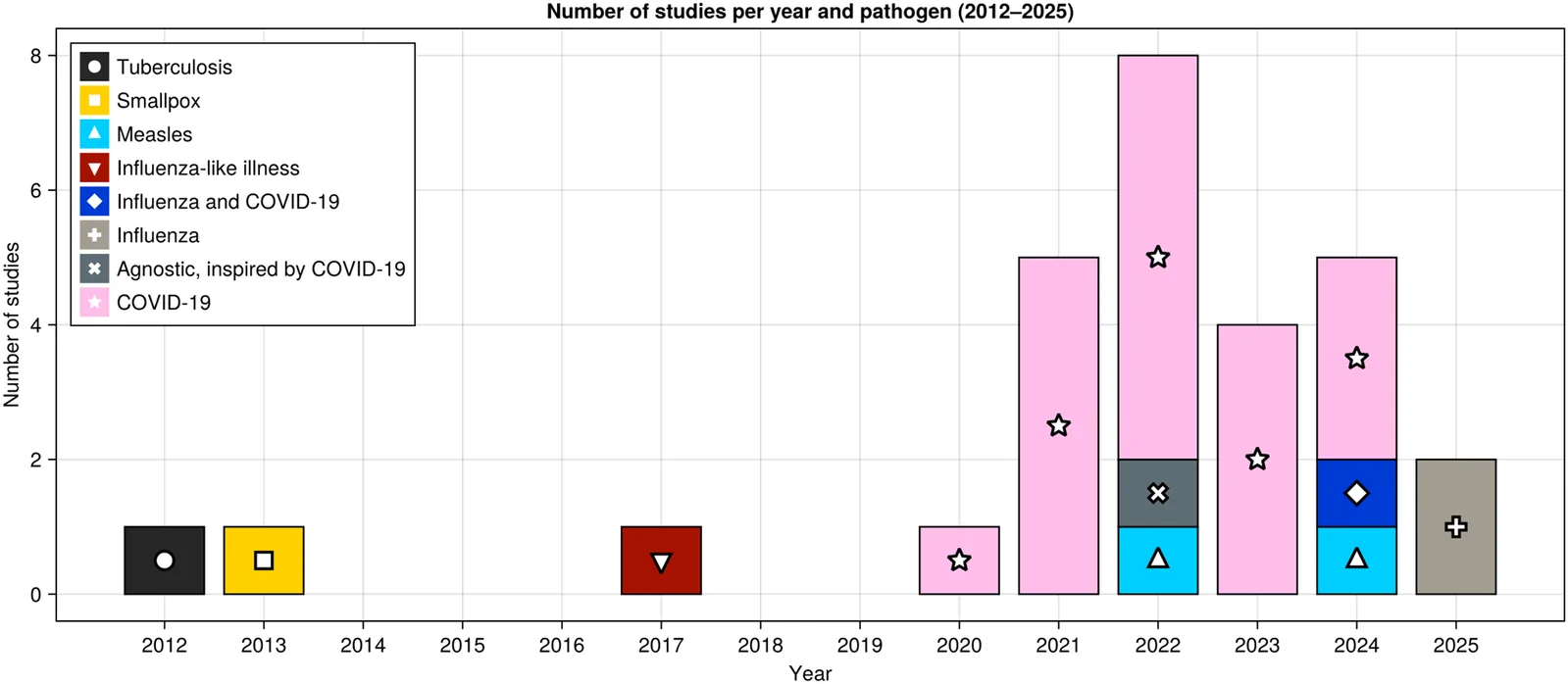
Publications included in the systematic review by year published and pathogen modelled. Symbols and colours represent tuberculosis (circle/black), smallpox (square/yellow), measles (arrow pointing upward/light blue), influenza-like illness (arrow pointing downward/dark pink), combined influenza and COVID-19 (diamond/dark blue), influenza (plus/light grey), COVID-19 (star/light pink), and pathogen-agnostic studies inspired by COVID-19 (x/dark grey). The height of each bar corresponds to the total number of studies published in that year. Figure 2. long description.

Figure 2 shows the surge in relevant papers published in 2020 or later (25 studies), most of which modelled COVID-19 (19 studies). Other studies modelled influenza (2), influenza and COVID-19 (1), tuberculosis (1), measles (2), influenza-like illness (1), smallpox (1), and an unspecified respiratory infectious disease (1). Only three papers were published prior to 2020 (2012, 2013, and 2017).

The most common purpose of the models identified in this review was to evaluate the effect of public health interventions, particularly vaccination, on disease inequities [27, 28, 37, 41, 43–46]. One study focused on the role of household structure in disease transmission, identifying household crowding as a key contributing factor for increased disease rates [48]. Spatial metapopulation models generally sought to explore the relationship between mobility and disease outcomes [26, 32, 33]. Ethnicity in these models was considered at a ZIP code or census block group (CBG) level, based on the proportion of different ethnicities in each spatial area. Barnes [24] compared calibrated state-level model parameters to socioeconomic indicators and diversity indices to investigate the relationship between these. Other studies demonstrated how different modelling assumptions lead to different results when modelling interventions. In particular, four studies demonstrated how different choices of the groups considered in the modelling, and different assumptions around assortativity in contact patterns, would affect the outcome of interventions [29, 30, 34, 36, 43].

Model design features, including population demographics, interaction heterogeneity, and parameter settings for the compartmental, metapopulation, and IBMs, are summarized in Tables 2–4.

**Table 2.** Model design features for the compartmental models that met the inclusion criteria Table 2. long description.

Author	Separate (unmixed) populations	Population compartments (demographic characteristics)	Population compartment mixing	Parameters that vary by ethnicity	Data sources for model construction	Data sources for calibration	Ethnic group comparisons in results
Barnes [24]	Each US state	None	Homogeneous mixing	NA	Census population counts	Case data	State-specific infection rates compared with consideration of diversity index. Also considered SES and access to healthcare in results
Datta et al. [28]	NA	Age (16 groups)	Age-structured contact matrix adapted from POLYMOD	NA	Population counts from health system administrative data (HSU)	Case, hospitalization, and mortality data at national level	Model rerun with different vaccination rates to determine the % of Māori deaths that could have been prevented with equitable vaccination
Elderd et al. [29]	Eight Jesuit Missions in southwestern US and Mexico, and five European/African outbreaks (New England, England, and Mauritius)	None	Homogeneous mixing	Initial immunity in the population, transmission rates, mortality rates, and variance.	Population estimates; existing immunity estimates from historic data	Monthly death counts	Compared parameter estimates for transmission rates, mortality rates, and variance for different outbreaks grouped by two ethnicity categories: European/African or Native-American
Engebretsen et al. [30]	NA	Ethnicity (2 hypothetical groups) and contact risk (2 groups)	Contact matrix where all individuals have the same number of contacts but varying the within- vs. between-group assortativity	A greater proportion of the population in ethnicity group B is high-risk	NA	NA	Calculating rate ratios and incidence ratios for different ethnic groups, and the calculated ethnicity coefficient in a Poisson regression fit on ethnicity and risk level. Showing how these are not representative of the underlying mechanisms in the model, and vary by R0, level of assortativity, and high/low risk ratios, even when you change nothing about ethnicity
Fleurantin et al. [31]	Each US state	Age (16 groups) and ethnicity (2 groups)	Age-structured contact matrix adapted from POLYMOD, with separate ethnicity contact matrix	Disease severity parameters. Different contact rates by ethnicity to represent higher levels of essential work and higher contacts for older adults in multi-generational houses. Time-varying R(T) from fitting to data	Census data for population size by age group and ethnicity (DAP vs. NDAP)	Total population case data and ethnicity-specific mortality data	Plots for each state comparing: reproductive number over time per ethnic group; number of people hospitalized and dead per ethnic group; or exposed, infected, recovered, and total cases over time per ethnic group
Kadelka et al. [34]	NA	Age (4 groups), workplace risk (2 groups), and ethnicity (2 groups)	Full age/ethnicity/workplace risk contact matrix for the USA, adapted from POLYMOD with different levels of ethnic assortativity and LC/HC job assortativity investigated	Vaccination rates under different strategies	Census population counts	Case data	Pairwise Spearman correlation plot showing trade-offs between deaths and cases in White/Asian vs. people of colour for the vaccination allocation strategy that minimized overall deaths. Two-dimensional Pareto frontier plots for vaccination allocation strategies showing these trade-offs. Table of deaths and cases by ethnicity for vaccination allocation strategies minimized deaths or cases for people of colour or White/Asian or total population
Klotz et al. [35]	Ethnicity groups (Immigrant, non-Indigenous Canadians, and Inuit)	None	Homogeneous mixing	Transmission rates	Census data for population and historic case data for initial conditions	Case data	Compare forecasted incidence rates from the independent Inuit, non-Indigenous Canadian, and Immigrant models
Ma et al. [36]	CBGs	Ethnicity (5 groups)	Ethnic group contact matrix with different levels of ethnic assortativity investigated	Transmission rates	Census population counts	Seroprevalence study results	Total contact rates from calibration, resulting cumulative incidence and incidence rate ratios over time for best-fit parameters
Owusu-Dampare and Bouchnita [37]	Ethnicity groups (Hispanic and non-Hispanic)	Age (6 groups)	Age-structured contact matrix adapted from POLYMOD	Hospitalization and mortality rates. Different contact matrices by ethnicity, due to increased contact rates for essential workers, indirectly increasing contact rates for Hispanic populations. Vaccination rates under different scenarios	Census population counts	Case data	Percentage differences for hospitalizations; hospitalization rates; and cumulative hospital admissions per ethnic group under different vaccination scenarios
Stafford et al. [43]	Ethnicity groups (White and BIPOC)	Age (5 groups)and ethnicity (2 groups)	Age-structured contact matrices adapted from US surveys and POLYMOD	Hospitalization and mortality rates. Different home and work contact matrices by ethnicity based on US contact surveys. Vaccination rates under different scenarios	Census population counts	Mortality data	Counterfactuals – equalizing disease outcome rates and then determining what percent vaccination is required for age/ethnicity groups to get equal outcome rates
Vino et al. [48]	Three independent communities (Indigenous Remote, Indigenous Urban, and non-Indigenous Urban)	Age (3 groups)	Age-structured household contact matrices derived from study surveys, with age-structured community contact matrices assuming fixed contacts per day and a proportionate mixing assumption using census age count data	Different household contact matrices for Indigenous vs. non-Indigenous and urban vs. remote Indigenous populations. All other parameters stayed the same	Bespoke household contact survey, census data for population counts, and constructing community contact matrices	NA	Compared effective R0, time-to-peak, peak prevalence, and cumulative infection proportion for the three different ethnicity/location categories: Indigenous Remote, Indigenous Urban, and non-Indigenous Urban

DAP, disproportionately affected population; NDAP, non-disproportionately affected population.

**Table 3.** Model design features for the metapopulation models that met the inclusion criteria Table 3. long description.

Author	Subpopulation spatial scale	Separate (unmixed) populations	Differences between subpopulations	Mixing within and between subpopulations	Ethnicity groups considered	Other drivers considered	Data sources for model construction	Data sources for calibration	Ethnic group comparisons in results
Chang et al. [26]	CBG	Ten (10 largest metropolitan areas in the USA)	Transmission rate varies by CBG	Mobility data linking CBGs together through mixing at POIs	Proportion non-White at CBG level	Median income of CBG	Census data, SafeGraph data	Case data at metro area level.	Calculated relative infection risk for ’non-white CBGs’. Compared mobility rates for the CBG in the top and bottom deciles for proportion White residents.
Chen et al. [27]	CBG	Nine (9 metropolitan areas in the USA)	IFR per CBG based on age structure. Vaccination rates per CBG under different strategies	Mobility data linking CBGs together through mixing at POIs, plus transmission at home	Ethnic-minority ratio calculated using ’proportion non-Hispanic white population’ at CBG level	Compare mean income, proportion essential workers, and proportion older adults at CBG level split by deciles in the results	Census data, SafeGraph data	Death data at metro area level	Correlations between ethnicity proportion and mobility rates, and deaths. Equity by ethnicity for four different vaccine prioritization strategies. Showing differences for different areas, and the need to consider the combination and intersectionality of demographic factors in a given area to understand ethnicity-specific impacts
Gyorgy et al. [32]	ZIP codes	One (Chicago, IL, USA)	Fit time-varying ’per trip risk’ per ZIP code	Daily trips within and between ZIP codes	ZIP codes categorized as one out of: majority White, majority Black, majority Latinx, and mixed	Compare with mean household size, median household income, percentage of individuals employed, percentage of individuals insured, percentage of overcrowded households, and the percentage of old buildings	Census data, SafeGraph data	Case data and testing data	Infection rates for different majority-ethnicity ZIP codes. Varying levels of homophily between ZIP codes with the same majority ethnicity to show contribution of homophily to case rate differences. Examining trade-offs with other risk factors
Hou et al. [33]	Custom regions formed from CBG-level mobility data cluster analysis	Two (Dane County and Milwaukee County, Wisconsin, USA)	Fit time-varying transmission rate within each region	Daily traffic flow between custom regions	Proportion of different ethnic groups (7) in each region	Age and type of business visited	Census data, SafeGraph data	Case data	Areas compared to determine any different trends in infection rates between predominantly White, Black, and Hispanic areas
Wang et al. [49]	ZIP codes	One (Greater Boston, MA, USA)		Mobility data counting movements within and between ZIP codes	ZIP codes categorized as one of: >80% White, >20% Latinx/Black, and mixed		Census data, SafeGraph data, vaccine data		Infection rates by ethnicity group

CBG, census block group; IFR, infection-fatality rate; POIs, points of interest.

**Table 4.** Model design features for the individual-based models that met the inclusion criteria Table 4. long description.

Author	Separate (unmixed) populations	Individual attributes	Consideration of relationships between attributes	Mixing between individuals	Differences by ethnicity	Other drivers considered	Data sources for model construction	Data sources for calibration/validation	Ethnic group comparisons in results
Arigbede [23]	Four regions	Age (6 groups), sex, ethnicity (8 groups), and social vulnerability index (4 groups)	Not stated.	Homogeneous mixing within each region.	Ethnicity-specific infection rates, recovery rates, and probabilities of ED visits		Health system administrative data (NSSP and CDC) for demographic statistics	Calibrated against ED visit data	Number of cases by ethnic group (not presented as rates, so difficult to compare)
Bilal et al. [25]	NA	Age (16 groups), race (7 groups), ethnicity (2 groups), and income	No. Attributes allocated based on independent distributions at ZIP code level	Synthetic interaction network where people interact at home, work, school, and community		Household size and presence of children at ZIP code level	Policy map for individual and household demographics. TomTom origin–destination flows across ZIP codes. Google Trends to modify behaviour through time	Calibrated against case data at ZIP code level	Ethnic-specific daily case counts and proportion of ethnicity groups in low vs. high transmission ZIP codes
Patel et al.[38]	NA	Age (5 groups), ethnicity (3 groups), and diabetes status	Not stated	Synthetic interaction network where people interact in home, work, school, and community	Ethnicity-specific infection, hospitalization, and mortality rates.	Time-varying workplace mobility estimates were calculated at rural/suburban/urban and income quintile category levels. Household size differences	Census population, household, and commuting data. SafeGraph mobility data to vary interaction parameters over time	Calibrated to state-level case and death data, validated against ethnicity-specific data	Ethnicity-specific infection, hospitalization, and mortality rates for different potential interventions
Quera-Bofarull et al. [40]	NA	Age (8 groups), ethnicity (5 groups), and socio-economic index (5 groups)	Some consideration	Synthetic interaction network where people interact at home, work, school, and community (JUNE)		Household size and structure in different area units	Data on population, households, workforce, schools, commuting patterns from the Office for National Statistics, UK. Commute data from the UK Department for Transport	Calibration against case, hospitalization, and death data using deep neural networks CalibNN	Proportion of population infected per ethnic groups, impact of different transmission context changes by ethnic groups
Rosenstrom et al. [41]	NA	Age (5 groups), ethnicity (4 groups), essential worker status, and diabetes status.	Essential worker status, diabetes status, and household size allocated based on distributions by ethnicity and age	Synthetic interaction network where people interact at home, work, school, and community (COVSIM.)	Differences in vaccine uptake and household size	Differences in disease progression by age and diabetes status	Census population, household, and work commuting data. SafeGraph mobility data to vary interaction parameters over time	Validated against ethnicity- and age-specific case, hospitalization, and death data	Age-adjusted infection and death rates per ethnic group for each scenario
Sivakumaar et al. [42]	Three US states	Age (4 groups), gender, and ethnicity (5 groups)	Age and ethnicity. But gender is assigned randomly	Simplified synthetic interaction network where people interact at home, work, school, and community (Hybrid COVASIM.)	Differences in disease progression, transmission rates, and vaccination rates		Census population and household data; Google mobility data to vary intervention parameters over time; vaccination data	Calibrated to state-level case data	Gini index for the exposed state and dead state for each ethnic group, each phase, and each intervention
Stafford et al. [44]	NA	Age (6 groups) and ethnicity (5 groups)	Yes.	Synthetic interaction network where people interact at home, work, school, and community (SynthPops COVASIM.)	Differences in disease progression and vaccination rates. Household size, workplace type (high-risk vs. low-risk), and school ethnicity assortativity		Census population, household, workforce, and school information	Calibrated to ethnicity-specific hospitalization, ICU, mortality, and vaccination rates	Symptomatic infection rate ratios and median percent difference in symptomatic infections from baseline for each ethnicity group compared to White
Starr and Kain [45]	Four US counties	Age (3 groups) and ethnicity (4 groups).	No. Attributes allocated independently	Not considered	Differences in vaccination rates		Census population counts	Validated against Westchester County case data	Regression to compare the relationship between the proportion of the population by ethnicity and the number of infections and deaths
Thakur et al. [46]	NA	Age (5 groups), gender, race (7 groups), and ethnicity (2 groups)	Yes.	Synthetic interaction network where people interact at home, work, school, and community. With extra detail on school contacts	Household size and household income. Differences in vaccination rates	Different vaccination rates by age	Census population, household, and workforce information, time-use surveys, travel surveys, education data, and vaccination data	Case data for 2018–2019 measles outbreak in NYC	Effect sizes for different vaccination scenarios per ethnic group. Wilcoxon’s signed-rank test to calculate effect size (more or less vulnerable) and compare vaccination rates and infection rates across groups
Thomas et al. [47]	NA	Age (9 groups), ethnicity (4 groups), and sex	Yes	Contact network built from spatial interaction functions based on distance, with individuals most likely to be linked to those in their household		Household size by census area	Census population counts and household size information	Validated against ethnicity-specific case data for San Francisco	Proportion of each ethnicity group infected
Williams et al. [50]	Eight US counties	Age (3 groups) and ethnicity (2 groups)	Yes	Synthetic interaction network where people interact at home, work, school, and community (FRED.)	Differences in vaccination rates	Number of children in the household	Census population counts, household, school, workplace, and commuting patterns	Calibrated to age group case rates	Case rate ratios for Black to White for equal vaccination rate simulation
Zhang et al. [51]	Three NZ health districts	Age (5 groups), ethnicity (4 groups), occupation, gender, and SES indicators	Not stated.	Synthetic interaction network where people interact at home, work, school, and community (JUNE-NZ)	Differences in disease progression and transmission rates, and vaccination rates	Household size and structure in different area units	Census population data, including household, workplace, school, and commuting. Health data on immunization history.	Calibrated against ethnicity-specific case data for 2019 measles outbreak in NZ in Manukau DHB	Percentage of measles infections by ethnicity group (showing results from simulation runs and empirical data)

CDC, Centers for Disease Control and Prevention; ED, emergency department; NSSP, National Syndromic Surveillance Program.

### Representations of population demographic heterogeneity

To represent real-world population demographics, most IBMs and compartmental models explicitly stratified the population by ethnicity and other demographic characteristics. Metapopulation models were the exception; these models all assumed homogeneous mixing within spatial areas, for example, ZIP codes, and relied on spatial heterogeneities to represent differences in disease transmission. Where ethnicity was considered, it was when analysing the results, and the ethnicity proportions or majority ethnicity in each spatial area was used. Investigating the representation of ethnicity in these models, we found that in general IBMs represented ethnicity at a finer granularity than compartmental or metapopulation models. IBMs typically categorized their populations into four or more ethnicity groups, whereas most compartmental models used two ethnicity groups only. The exception being the compartmental model by Ma et al. [36], which considered five ethnicity groups; however, the trade-off was that they included no other demographic factors. No models included consideration of people with multiple ethnicities.

Models that include variables such as age, SES, household structure, or workplace type in addition to ethnicity are incredibly valuable because these are some of the key drivers of the observed disparities in disease burden [2, 6]. Of the other demographic factors included, age was the most common, with most IBM and compartmental models (18 out of 23) separating the population by age groups. Household structure was also important, with ten IBMs explicitly representing different household sizes and four compartmental models adjusting their contact matrices to reflect different household size distributions for subpopulations. For COVID-19 in particular, workplace risk was often considered, with four models directly including a workplace risk attribute or compartment, and five other models adjusting their contact structure based on workplace risk. Other less common population attributes included sex/gender, socio-economic status (SES), social vulnerability, and health risk. These extra factors were often only included in IBMs, as they would lead to a prohibitive number of compartments in a compartmental or metapopulation model.

In our review, we also assessed whether the covariance or intersectionality of different attributes was well represented. We found that in many cases this was difficult to ascertain, with only a few papers explicitly describing which covariates they captured. Furthermore, in several models, individual attributes were allocated independently or only using spatial-level heterogeneities. For example, many IBMs reproduced the household size distribution at the spatial area unit but did not consider differences in household size by other factors such as income, age, or ethnicity. If the relationships between factors are not well represented, then estimations of the impact of interventions related to these factors on different ethnic groups may not be reliable.

### Representations of interaction heterogeneity

Different model types vary widely in how much interaction heterogeneity can be represented. Network-based IBMs are able to represent individual contact patterns, whereas compartmental models use contact matrices to capture mixing within and between groups, and spatial metapopulation models capture mixing within and between spatial areas.

All five metapopulation models used mobility data from SafeGraph to estimate mixing rates at the level of CBGs [26, 27], ZIP codes [32, 49] and custom aggregations of CBGs derived from cluster analysis of mobility patterns [33]. In these models, any relationships between ethnicity or other demographic factors and contact rates are only captured indirectly through the demographic composition of the different spatial areas, and the high levels of spatial assortativity in contact patterns.

IBMs are able to capture the most detail in contact patterns for disease transmission, and this is reflected in the models we reviewed. Nine of the 12 IBMs constructed detailed interaction networks to explicitly link individuals through shared households, schools, workplaces, and community interactions [25, 38, 40–42, 44, 46, 47, 51]. These models used official statistics data (e.g. census) to construct representative homes, workplaces, and schools. Then, to link individuals to workplaces and schools, six IBMs used commuting data from official statistics, one used the TomTom OD analysis engine [25], and the remainder used location- or distance-based heuristics. One IBM constructed an interaction network using a spatial interaction function for most contacts, but with differences in the household size distribution at census tract level [47]. In contrast, two IBMs assumed homogeneous population mixing, with Starr and Kain [45] using a random walk to determine agent interactions, and Arigbede [23] assumed the population within each geographic area was well-mixed.

Five compartmental models incorporated ethnicity-specific differences in age-structured mixing patterns. Three studies extended the European POLYMOD age-structured contact matrices [52] to include race/ethnicity and high/low workplace risk [34] compartments, including considerations such as differences in household size, inter-generational households, and proportion of essential and frontline workers. These three studies then used varying assumptions on ethnicity assortativity to generate the necessary within- and between-ethnicity contact matrix elements. Two studies constructed different age-structured contact patterns for different ethnicities, taking into account differences in household structure [48], or workplace contact rates [37] by ethnicity, and ran the populations independently.

Ma et al. [36] did not stratify the population by age, only ethnicity, and constructed an ethnicity-structured contact matrix using ethnic assortativity assumptions, and Engebretsen et al. [30] explored varying degrees of ethnic assortativity on the resulting disease outcomes across hypothetical ethnic groups. In contrast, Datta et al. [28] used only an age-structured contact matrix, and three compartmental models [23, 24, 35] assumed homogeneous mixing with no separate demographic groups.

### Disease parameter settings

Several studies incorporated ethnicity-specific disease transmission and progression parameters by ethnicity. Focusing initially on models that varied transmission parameters, we found that two compartmental models varied disease susceptibility or infectivity by ethnicity to best reproduce empirical trends [35, 36]. Similarly, four IBMs varied either the infectivity or susceptibility of individuals based on their ethnicity [23, 38, 42, 51].

In addition to disease transmission parameters that varied by ethnicity, we also consider models that vary transmission parameters spatially, as these differences lead indirectly to differences by ethnicity due to spatial segregation. For example, Elderd et al. [29] found that setting a different basic reproduction number R0 for different populations best reproduced empirical smallpox patterns. In more recent COVID-19 models, most metapopulation models, and at least two of the IBMs, varied transmission rates by spatial areas to fit case data. Two IBMs [38, 41] and all metapopulation models used data from SafeGraph (an aggregated and anonymized mobile phone mobility dataset) and/or Google mobility trends to estimate time-varying mobility rates for different spatial areas in response to lockdown measures.

Other disease progression parameters, including rates of hospitalization and mortality, were varied by ethnicity in four compartmental models [29, 31, 37, 43], and in five IBMs [23, 38, 42, 44, 51].

A key driver of differences in respiratory disease inequities is differences in vaccination rates. Depending on the disease and the vaccine, vaccination or prior immunity can affect both disease transmission and disease progression. Four compartmental models [29, 34, 37, 43], one metapopulation model [27], and six IBMs [42, 44–46, 50, 51] included differences in vaccination rates or prior immunity by ethnicity, either from pre-existing data or using estimated vaccination rates under different scenarios as a key element of the study. Datta et al. [28] used a single age-structured population, so could not set different vaccination rates for different ethnic groups. Instead, they considered different counterfactual scenarios with vaccination rates for the whole population set to the levels seen for the Māori population or the non-Māori population.

### Model calibration and validation

Most IBMs reported calibration or validation using empirical data, but it was often not clear which parameters were adjusted and whether these were ethnicity specific. Notable exceptions include five studies that calibrated to [41, 44, 47, 51] or validated against [38] empirical ethnicity-specific case, hospitalization, ICU, and mortality data.

Advanced methods, such as machine learning and ensemble data assimilation, were used to calibrate parameters to overall case or fatality data in several IBMs and one metapopulation model [23, 33, 40, 51]. Other metapopulation models calibrated parameters for each homogeneous spatial unit to empirical case or fatality data [26, 27, 32, 49].

Several studies considered empirical data biases and limitations in their model design. Fleurantin et al. [31] and Stafford et al. [43] calibrated only to ethnicity-specific COVID-19 mortality data due to the known under-representation of some ethnic groups in case data. Fleurantin et al. [31] also restricted their model to the ten spatial regions (US states) where ethnicity information was at least 93% complete. Ma et al. [36] used seroprevalence survey data which had higher quality ethnicity information, and less under-reporting bias, than case and hospitalization data. Datta et al. [28] performed sensitivity analyses to account for the potential under-representation of the Māori ethnic group in the administrative dataset.

### Discussion of inequities

Framing and interpretation of differences in outcomes by ethnicity varied across studies included in this review. Most studies state differences as inequitable, link them to unfair and unequal access to the social determinants of health, relate findings back to potential impacts on real-world inequities, and discuss data and model design limitations that could bias the results.

In contrast, other studies described differences by ethnicity descriptively, without framing the observed differences as unfair or avoidable. These studies generally also had limited discussions of the underlying structural drivers of inequities. Notably, several large and widely used simulation frameworks, including JUNE [40, 51], CitySEIRCast [25], COVASIM [38, 41], and FRED [50], incorporated detailed demographic structure but presented ethnic differences in publications as descriptive only.

### Bias assessment

Potential sources of bias for the included studies are summarized in Table 5. While there are no formal frameworks for evaluating bias in mathematical models, we selected five key themes as documented in Grading of Recommendations Assessment, Development and Evaluation (GRADE) [53]. Of the 28 included studies, 20 discussed biases from empirical data used in models, and 25 discussed limitations of the model framework used. Only 14 studies published all code and datasets required to reproduce the model and analyses, but an additional 4 studies noted data were available upon request. Most studies stated funding source (23/28) and conflicts of interest (20/28). Transparency on model and data bias and limitations ensures the findings are credible and appropriate for use in informing public health strategies.

**Table 5.** Bias assessment for studies included in the systematic review Table 5. long description.

Author	Authors note biases from empirical data	Authors note model limitations	Data and code availability	Funding identified	Declare conflicts of interest
Arigbede [23]	Yes	Yes	No code or data available	No	No (thesis)
Barnes [24]	No	No	No code or data available	No	No (thesis)
Bilal et al. [25]	Yes	Yes	Data available upon reasonable request, code not available	Yes	Not stated
Chang et al. [26]	Yes	Yes	Code and data are open access	Yes	None to declare
Chen et al. [27]	No	Yes	Code and data are open access	Yes	None to declare
Datta et al. [28]	Yes	Yes	Code and data are open access	Yes	Declared
Elderd et al. [29]	Yes	Yes	No code or data available	Yes	Not stated
Engebretsen et al. [30]	Yes	Yes	Code is open access; no data were used for the study	Yes	None to declare
Fleurantin et al. [31]	Yes	Yes	Code and data are open access	Yes	Declared
Gyorgy et al. [32]	Yes	Yes	Code and data are open access	Yes	None to declare
Hou et al. [33]	No	Yes	Code and data are open access	Yes	None to declare
Kadelka et al. [34]	Yes	Yes	Code and data are open access	Yes	None to declare
Klotz et al. [35]	Yes	Yes	No code or data available	Yes	Declared
Ma et al. [36]	Yes	Yes	Code and data are open access	Yes	Declared
Owusu-Dampare and Bouchnita [37]	Yes	Yes	Model equations and data are available	Yes	Not stated
Patel et al. [38]	Yes	Yes	No code or data available	Yes	Declared
Quera-Bofarull et al. [40]	No	Yes	Code and data are open access	Yes	Not stated
Rosenstrom et al. [41]	Yes	Yes	Data available upon reasonable request, code not available	Yes	Declared
Sivakumaar et al. [42]	Yes	Yes	Data available upon reasonable request, code not available	No	None to declare
Stafford et al. [43]	Yes	Yes	Code and data are open access	Yes	None to declare
Stafford et al. [44]	Yes	Yes	No code or data available	Yes	None to declare
Starr and Kain [45]	No	Yes	No code or data available	No	Not stated
Thakur et al. [46]	Yes	Yes	Data available upon reasonable request, code not available	Yes	None to declare
Thomas et al. [47]	No	No	Code and data are open access	Yes	None to declare
Vino et al. [48]	Yes	Yes	Code and data are open access	Yes	Declared
Wang et al. [49]	No	Yes	Some data available, code not linked	Yes	None to declare
Williams et al. [50]	Yes	No	No code or data available	Yes	Declared
Zhang et al. [51]	No	Yes	No code or data available	No	Not stated

Nearly all (27/28) studies used census or administrative data for building their populations. These datasets introduce ingrained data biases into models, as ethnicity may be misclassified, missing, or overly aggregated, with differential effects across groups [54]. For example, 53% of migrant workers in the USA are undocumented and not captured in census data [41], while in NZ, ethnicity misclassification is greater for Māori, Pacific, and Middle-Eastern/Latin American/African populations despite recent improvements [55]. Additionally, metapopulation models in this review all relied on anonymized mobility data, which lack demographic information, may misrepresent some populations, and are unavailable or inappropriate in certain settings [56]. Very few studies explicitly acknowledged these data limitations, highlighting the need for methods that account for data bias during model design and calibration [3].

The majority of the studies were US-based COVID-19 models (see Table 1). Despite low ethnicity reporting for COVID-19 case and mortality data, US-based studies typically used this data to calibrate models [57]. To mitigate data limitations, one study restricted analyses to states with adequate data [31], two relied on mortality data that had better coverage [31, 43], and one instead used seroprevalence estimates [36]. While seroprevalence studies provide better estimates of cumulative infections, they typically lack ethnic and demographic detail, which can be limiting for models focused on investigating inequities, unless otherwise accounted for [39, 58, 59].

## Discussion

We identified 28 mathematical epidemiology studies that model infectious respiratory disease transmission and report their results by ethnicity. We found that the COVID-19 pandemic was a key catalyst for improving mathematical modelling of ethnic inequities; only 3 papers out of the 28 were published prior to 2020. These 28 publications represent a very small proportion of the totality of mathematical respiratory disease modelling literature, suggesting that mathematical modelling of ethnicity-related differences in infectious respiratory disease is an under-studied area.

An important point to note was that there were many studies which did not meet the inclusion criteria, despite consideration of some of the key drivers of different infection risks by ethnicity, for example, age structure, household size, income, as they did not take the final step of analysing what the results would be for different ethnic groups. Although this would require consideration of how to add ethnicity as an attribute in an IBM, how to stratify a compartmental model by ethnicity, or how to scale the results based on ethnicity distributions, it would be a valuable step in advancing the field.

When comparing mathematical modelling approaches, we found that IBMs offer greater flexibility than compartmental models for incorporating individual-level heterogeneity. In contrast, compartmental models generally used highly aggregated demographic groups, which minimized complexity and data requirements. However, even for IBMs, which could technically have very large numbers of different ethnicity categories, we found that there is often a trade-off between aggregating dissimilar groups and having sufficient data to populate the model. Relying on highly aggregated categories such as Black/White [50] obscures inequities affecting other groups. Similarly, major ethnicity categories typically include culturally diverse and distinct ethnic groups within them. The appropriate levels of aggregation and ethnicity categorizations to use for a model will depend on the research question and whether data are sufficient for reproducing the differential drivers of inequities for different groups. The approach of Fleurantin et al. [31], which defined the ethnicity grouping for the ‘Disproportionately Affected Population’ state by state, provides one example of how to let the context inform the appropriate categorization.

An important gap identified in this review was that many of the studies did not ensure that they captured the relationships between the individual attributes they included. For example, differences in household sizes for different ages and ethnic groups. In general, representing the intersectionality of multiple attributes in a heterogeneous population is non-trivial, but recent research on the development of synthetic populations shows how to represent real-world demographic structures while mitigating privacy risks and data gaps [60, 61]. Open access and well-evaluated methods for developing synthetic populations specific to the research question are required, and their use must be guided by Indigenous data sovereignty principles, including understanding data limitations and how these affect Indigenous groups.

The importance of contact structures for disease transmission is well established [18], and a number of the studies identified in this review explicitly demonstrated that including (or not including) contact pattern assortativity, including by ethnicity, type of work, and location, qualitatively changed the relative impacts of policies assessed in the modelling [30, 34, 36, 43]. Most compartmental models rely on contact matrices derived from surveys such as the European POLYMOD survey [52], as contact survey data are not available for many countries. However, existing international surveys are unlikely to appropriately represent some ethnic groups, especially minority ethnicities [62], and may not be applicable to different cultural contexts, such as the USA. Additionally, most of the contact matrices derived from survey data typically only capture the average contacts by age, and either do not include mixing between ethnic groups or rely on simple heuristics. For IBMs, constructing representative social contact networks is challenging and intensive. Several studies reduced resource requirements by adapting existing platforms such as JUNE [40, 51], CitySEIRCast [25], COVASIM [38, 41], and FRED [50]. However, the commonly used data sources for these networks (census, administrative, survey, and mobility data) each come with distinct gaps and biases [63], and often simple heuristics are used for spatial and community assortativity.

When seeking to capture ethnic inequities of respiratory disease spread, it is worth considering which dimensions are most important to represent in population structures, and hence in contact matrix data, for mathematical epidemiology [64–66]. Country-specific surveys that stratify by these important dimensions would enhance disease models [5]. The recent Reconnect [67] cross-sectional contact survey and the work in Vino et al. [48] provide examples of how surveys can be used to inform ethnic-specific interaction patterns.

In addition to the contact structure, many models rely on simplifying assumptions to capture other effects on disease transmission [19]. A number of the models in this review varied transmission rates by ethnicity (or spatial area) to calibrate to empirical case data. In this way, modellers were using ethnicity as a proxy for the actual underlying drivers of the higher infection rates. Although ethnicity may be predictive of health outcomes, it is not typically a direct driver; fitting ethnicity-specific parameters to the observed case data risks essentializing ethnicity as the ‘cause’ of the differential outcomes and can obscure the underlying mechanisms that drive inequities. Additionally, it means that the evaluation of any interventions or policies that act on unrepresented underlying drivers can be unreliable [2, 6]. Finally, the same structural factors that influence infection risk may also influence case reporting; hence, calibrating to case data can lead to a biased model.

Where possible, modellers should avoid using ethnicity as a proxy for the drivers of differences in respiratory disease infections in models. By setting non-ethnicity-specific parameters for variables that drive ethnic inequities, modellers can avoid essentializing ethnicity as a causative factor and can better represent the real-world mechanisms of disease transmission and impacts of interventions. This, alongside careful interpretations of results, data sources, and model limitations within the context of health inequities, will reduce the risk of deficit framing [68] and further marginalizing vulnerable ethnic groups [2, 69, 70].

Choosing what drivers to include in a specific model requires a nuanced understanding of what drives disease in the population of interest and will depend on the research questions that the model is trying to answer. For example, when modelling the effect of school closures on COVID-19 rates by age and ethnicity during lockdown, it may be necessary to include a multigenerational household variable for the model population, where older adults become protected through this intervention. Findings from epidemiological data [5] and existing modelling studies can help identify important factors or knowledge gaps to address. There is no single ‘best’ model, and there is high value in methodological pluralism [71].

While it can be tempting to model as much as possible so that factors are not accidentally missed, this risks overcomplicating models and makes calibration and validation more challenging. When selecting attributes and variables to include, modellers should strive to include only the essential factors for the dynamics being studied. In addition, modellers should consider their choices and language used to ensure that the modelling uplifts communities and avoids deficit framing [68].

One final recommendation for mathematical epidemiological modelling studies is to strengthen the language regarding health inequities, which acknowledges the inequitable nature of differences and encompasses the ethical imperative to address unfairly distributed resources necessary for good health. This shifts the focus required for improving differences from the vulnerable population and onto the systemic drivers that have led to these differences [72, 73]. Although health inequities were the central theme across most of the 28 studies included in this review, some studies continue to describe avoidable differences in outcomes by ethnic groups as inequalities and disparities. While many health outcomes are significantly more prevalent for a particular group, such as dementia in older people, or ovarian cancer in females, these differences, or ‘disparities’, are not necessarily avoidable but are due to biological factors, whereas many infectious diseases and other chronic and acute conditions that affect lower socio-economic and some ethnic groups at higher rates are unfair and avoidable. An appropriate use of ‘inequities’ creates more transparent research by unmasking social determinant pathways that drive these differences, making it easier to identify opportunities for interventions targeting the root cause of the inequities.

Ethnic inequities arise from the social and political contexts that drive unfair distributions of social determinants of health, including income, employment type, and housing quality. The mechanisms in which these factors compound, intertwine, and feedback are complex and unpredictable, and the limited ability to disentangle these effects has implications for designing mathematical models. Model design features become easier to trade-off between, once modellers fully commit to addressing health inequities.

## Limitations of this review

While this review included published and unpublished papers, other relevant modelling literature (e.g. government and research reports) may have been missed. Additionally, some models may have been capable of reporting ethnicity-specific results but did not, introducing potential bias if papers only reported ethnicity-specific results when differences were observed. Studies focusing on a single ethnic group were excluded but may have used relevant modelling methods for representing ethnic groups who experience inequities. Further, only 10% of title-screen exclusions were checked, increasing the risk of exclusion bias. Given the breadth of the field of mathematical epidemiology, we restricted the review to mechanistic models of respiratory pathogen transmission. Many other types of statistical and mathematical models exist and are valuable for strengthening our understanding of respiratory infectious disease and its impact on ethnic and marginalized groups, and for informing model design and parameter choices for mechanistic transmission models, but they are not the focus of this review. Methodological pluralism is encouraged as it strengthens our understanding and improves confidence in capturing ethnic inequities [71].

Most studies were US based, which may mean that the ethnicity-specific findings from the model results may not be generalizable outside of US settings. For example, while the USA and NZ both have significant health inequities for ethnic groups, each has different ethnic compositions, ways of defining race and ethnicity, and drivers for increased disease risk (such as greater health care access barriers in the USA) [74]. Nonetheless, we believe the findings related to methods and model design from this review are generalizable outside of the USA.

## Conclusions

This review shows that, although we did not find a large number of examples, ethnic inequities in respiratory infectious disease burden can be meaningfully represented in mathematical models. To do this well requires that factors such as population heterogeneity, differences in interaction structure, interaction assortativity, data limitations, and contextual interpretation are carefully considered and transparently incorporated into model design. A fundamental application of infectious disease models is evaluating public health interventions. While it can be difficult to appropriately model factors that can lead to different infection outcomes by ethnicity in models, doing so is essential for evaluating the differential impacts of potential policies. Without appropriate representation of different ethnic groups, and the mechanistic factors known to influence differential exposure, disease transmission, and disease burden, models are unlikely to accurately assess the relative impact of interventions across population groups (as shown in Refs. [30, 34, 36, 43]). There is a clear need for better data on the heterogeneity and assortativity of contact patterns within and between different communities. Studies that incorporate population and contact heterogeneities and context-specific interpretations are better positioned to explore how policy choices may affect different population groups [6].

Considering ethnic inequities in mathematical models is complex but essential if researchers and policymakers want to continue to use these models to gain insights that support strategic public health planning. Ignoring equity risks, misrepresenting transmission mechanisms, and developing models that are not accurate reflections of the unique realities of all groups can potentially widen inequities when applied in real-world settings. As mathematical models are increasingly used to inform public health action, careful attention to incorporating and interpreting ethnic inequities, including transparency on data limitations and appropriate application of models, is essential to ensure that models can be used alongside other epidemiological studies to provide evidence for public health strategies that will reduce inequities and improve health for the most vulnerable populations.

## Supporting information

10.1017/S0950268826102118.sm001 Kumbaroff et al. supplementary material 1 Kumbaroff et al. supplementary material

10.1017/S0950268826102118.sm002 Kumbaroff et al. supplementary material 2 Kumbaroff et al. supplementary material

## Data Availability

Data extracted from the studies included in this review are provided in Tables 1 and 2. No other data were used for this systematic review.

## Supplementary material

The supplementary material for this article can be found at http://doi.org/10.1017/S0950268826102118.

## Long descriptions

### Figure 1. Long description

The flowchart is organized into three vertical phases: Identification, Screening, and Included.

1. Identification Phase:

- Top-left box: Records identified from Wiley (n = 511), Cochrane (n = 20), E B S C O (n = 541), P R O Q U E S T (n = 751), and Scopus (n = 1545). Total N = 3368.

- Top-right box (exclusion): Records removed before screening due to duplicate records removed by Endnote (n = 1123) and manually (n = 130). Total: 1253.

2. Screening Phase:

- Second box down (left): Records screened (n = 2115).

- Second box down (right): Records excluded (n = 1818).

- Third box down (left): Reports sought for retrieval (n = 297).

- Third box down (right): Reports not retrieved (n = 11).

- Fourth box down (left): Reports assessed for eligibility (n = 286).

- Fourth box down (right): Reports excluded for specific reasons: No results by ethnic group (n = 172), Compares geographically separated populations (n = 34), Does not model respiratory infectious disease dynamics (n = 33), Restricted to vulnerable population only (n = 17), Review article (n = 9), and Animal model only (n = 1).

3. Included Phase:

- Bottom-left box: Final studies included in review from Databases (n = 17) and Citation search (n = 11). Total = 28.

Navigate back to Figure 1..

### Table 1. Long description

The table contains seven columns: Author, Geographical setting, Model type, Disease and immunity states, Deterministic vs. stochastic, Pathogen, and Model purpose.

Key entries include:

* Arigbede: USA, I B M, 6 states, Stochastic, Influenza and COVID-19, predicting health service utilization.

* Barnes: USA, C M, 3 states, Deterministic, COVID-19, examining social determinants of health.

* Bilal et al.: Hillsborough, Florida, USA, I B M, 14 states, Stochastic, COVID-19, city-scale simulation.

* Chang et al. and Chen et al.: USA metropolitan areas, M M, 4 states, Stochastic, COVID-19, focusing on mobility and equity.

* Datta et al.: N Z, C M, 14 states, Deterministic, COVID-19, vaccination counterfactuals.

* Elderd et al.: Multiple international locations, C M, 4 states, Deterministic, Smallpox, R 0 differences.

* Engebretsen et al.: Hypothetical, C M, 3 states, Stochastic, Agnostic/COVID-19, community-level risk.

* Fleurantin et al.: USA, C M, 12 states, Deterministic, COVID-19, pandemic response evaluation.

* Kadelka et al.: USA, C M, 20 states, Deterministic, COVID-19, ethnic and occupation-based homophily.

* Klotz et al.: Quebec, Canada, C M, 5 states, Deterministic, Tuberculosis, long-term incidence forecasting.

* Quera-Bofarull et al.: London, U K, I B M, 9 states, Stochastic, COVID-19, city-scale simulation.

* Stafford et al.: Oregon, USA, C M, 18 states, Deterministic, COVID-19, minimizing deaths vs. years of life lost.

* Thakur et al.: Virginia, USA, I B M, 5 states, Stochastic, Measles, vaccination disparities.

* Zhang et al.: N Z, I B M, 3 states, Stochastic, Measles, machine learning calibration.

Abbreviations defined below the table: C M stands for compartmental model, I B M stands for individual-based model, and M M stands for metapopulation model.

Navigate back to Table 1..

### Figure 2. Long description

The x-axis represents the Year from 2012 to 2025. The y-axis represents the Number of studies from 0 to 8.

Data trends by year:

* 2012: 1 study on Tuberculosis.

* 2013: 1 study on Smallpox.

* 2014 to 2016: 0 studies.

* 2017: 1 study on Measles.

* 2018 to 2019: 0 studies.

* 2020: 1 study on C O V I D-19.

* 2021: 5 studies on C O V I D-19.

* 2022: 8 studies total, consisting of 1 Smallpox, 1 Influenza, and 6 C O V I D-19.

* 2023: 4 studies on C O V I D-19.

* 2024: 5 studies total, consisting of 1 Smallpox, 1 Influenza-like illness, and 3 C O V I D-19.

* 2025: 2 studies on Tuberculosis.

The legend on the right identifies the pathogens: Tuberculosis (black), Smallpox (lightest gray), Measles (light gray), Influenza-like illness (medium-light gray), Influenza and C O V I D-19 (medium gray), Influenza (dark gray), Agnostic inspired by C O V I D-19 (darker gray), and C O V I D-19 (darkest gray). Note that while the caption mentions colors, the provided image is in grayscale.

Navigate back to Figure 2..

### Table 2. Long description

The table contains eight columns: Author, Separate (unmixed) populations, Population compartments (demographic characteristics), Population compartment mixing, Parameters that vary by ethnicity, Data sources for model construction, Data sources for calibration, and Ethnic group comparisons in results.

Key entries include:

* Barnes: Focuses on each U S state with homogeneous mixing and state-specific infection rates compared with diversity index.

* Datta et al.: Uses 16 age groups and age-structured contact matrices from POLYMOD to determine preventable M ā ori deaths via equitable vaccination.

* Elderd et al.: Covers Jesuit Missions and European/African outbreaks, varying initial immunity and transmission rates by ethnicity.

* Engebretsen et al.: Uses hypothetical groups to show how rate ratios vary by R 0 and assortativity.

* Fleurantin et al.: Models U S states with 16 age groups and 2 ethnicity groups (D A P and N D A P), varying disease severity and contact rates.

* Kadelka et al.: Includes age, workplace risk, and ethnicity, using Pareto frontier plots to show trade-offs in vaccination strategies between White/Asian and people of color.

* Klotz et al.: Compares Immigrant, non-Indigenous Canadians, and Inuit populations with varying transmission rates.

* Ma et al.: Uses 5 ethnicity groups and C B G s to investigate ethnic assortativity and incidence rate ratios.

* Owusu-Dampare and Bouchnita: Compares Hispanic and non-Hispanic populations across 6 age groups, varying hospitalization and mortality rates.

* Stafford et al.: Compares White and B I P O C populations, equalizing disease outcome rates to determine required vaccination percentages.

* Vino et al.: Compares Indigenous Remote, Indigenous Urban, and non-Indigenous Urban communities using bespoke household contact surveys.

Navigate back to Table 2..

### Table 3. Long description

The table consists of 10 columns and 5 data rows.

Columns from left to right:

1. Author

2. Subpopulation spatial scale

3. Separate (unmixed) populations

4. Differences between subpopulations

5. Mixing within and between subpopulations

6. Ethnicity groups considered

7. Other drivers considered

8. Data sources for model construction

9. Data sources for calibration

10. Ethnic group comparisons in results

Data Rows:

- Chang et al.: Scale is C B G. Populations include 10 largest U S A metro areas. Transmission varies by C B G. Mixing via mobility at P O Is. Ethnicity is proportion non-White. Other driver is median income. Sources include Census and SafeGraph. Calibrated with metro case data. Results compare infection risk for non-White C B Gs.

- Chen et al.: Scale is C B G. Populations include 9 U S A metro areas. I F R and vaccination rates vary. Mixing via mobility and home transmission. Ethnicity is proportion non-Hispanic White. Other drivers include income, essential workers, and older adults. Sources include Census and SafeGraph. Calibrated with metro death data. Results show correlations between ethnicity, mobility, and deaths.

- Gyorgy et al.: Scale is Z I P codes. Population is Chicago, I L. Fit per-trip risk per Z I P. Mixing via daily trips. Ethnicity categorized as majority White, Black, Latinx, or mixed. Other drivers include household size, income, employment, and housing quality. Sources include Census and SafeGraph. Calibrated with case and testing data. Results examine infection rates and homophily.

- Hou et al.: Scale is custom regions from C B G mobility clusters. Populations are Dane and Milwaukee counties, W I. Fit time-varying transmission. Mixing via daily traffic flow. Ethnicity is proportion of 7 groups. Other drivers are age and business type. Sources include Census and SafeGraph. Calibrated with case data. Results compare trends in predominantly White, Black, and Hispanic areas.

- Wang et al.: Scale is Z I P codes. Population is Greater Boston, M A. Mixing via mobility between Z I P codes. Ethnicity categorized as greater than 80 percent White, greater than 20 percent Latinx or Black, or mixed. Sources include Census, SafeGraph, and vaccine data. Results show infection rates by ethnicity group.

Navigate back to Table 3..

### Table 4. Long description

The table contains 10 columns: Author, Separate (unmixed) populations, Individual attributes, Consideration of relationships between attributes, Mixing between individuals, Differences by ethnicity, Other drivers considered, Data sources for model construction, Data sources for calibration/validation, and Ethnic group comparisons in results.

Key entries include:

* Arigbede: Models 4 regions with 8 ethnicity groups. Uses homogeneous mixing within regions and ethnicity-specific infection rates. Calibrated against E D visit data.

* Bilal et al.: Uses 16 age groups and 7 race groups. Features a synthetic interaction network (home, work, school, community). Calibrated against ZIP code case data.

* Patel et al.: Focuses on 3 ethnicity groups and diabetes status. Includes ethnicity-specific hospitalization and mortality rates. Validated against ethnicity-specific data.

* Quera-Bofarull et al.: Uses the J U N E synthetic interaction network for 5 ethnicity groups. Calibrated using deep neural networks (Calib N N).

* Rosenstrom et al.: Uses the C O V S I M network. Considers essential worker status and diabetes. Validated against age and ethnicity-specific case and death data.

* Sivakumaar et al.: Models 3 U S states using a Hybrid C O V A S I M network. Considers differences in disease progression and vaccination rates.

* Stafford et al.: Uses SynthPops C O V A S I M. Includes school ethnicity assortativity and workplace risk levels. Calibrated to ethnicity-specific I C U and mortality rates.

* Starr and Kain: Models 4 U S counties with 4 ethnicity groups. Focuses on differences in vaccination rates. Validated against Westchester County case data.

* Thakur et al.: Models N Y C measles outbreak. Includes extra detail on school contacts and household income. Uses Wilcoxon signed-rank tests for comparisons.

* Thomas et al.: Models San Francisco using spatial interaction functions based on distance. Validated against ethnicity-specific case data.

* Williams et al.: Uses the F R E D network for 8 U S counties. Compares Black to White case rate ratios for equal vaccination simulations.

* Zhang et al.: Uses J U N E-N Z for 3 New Zealand health districts. Includes S E S indicators and immunization history. Calibrated against 2019 measles outbreak data.

Navigate back to Table 4..

### Table 5. Long description

The table consists of six columns: Author, Authors note biases from empirical data, Authors note model limitations, Data and code availability, Funding identified, and Declare conflicts of interest.

Key data points include:

* Arigbede: Yes for biases and limitations; No code/data; No funding; No conflict (thesis).

* Barnes: No for biases and limitations; No code/data; No funding; No conflict (thesis).

* Bilal et al.: Yes for biases and limitations; Data on request, no code; Yes funding; Not stated conflict.

* Chang et al., Datta et al., Fleurantin et al., Gyorgy et al., Kadelka et al., Ma et al., Stafford et al. (44), and Vino et al.: All report Yes for biases and limitations, and provide open access to code and data.

* Chen et al., Hou et al., Quera-Bofarull et al., Starr and Kain, Wang et al., and Zhang et al.: These authors did not note biases from empirical data but did note model limitations.

* Thomas et al.: No for biases and limitations; Open access code and data; Yes funding; None to declare.

* Williams et al.: Yes for biases; No for limitations; No code/data; Yes funding; Declared conflict.

* Studies by Arigbede, Barnes, Sivakumaar et al., Starr and Kain, and Zhang et al. did not identify funding.

Navigate back to Table 5..
