# Supplementary material for: Ethnic inequities in mathematical models for respiratory infectious disease: A systematic review
Source: Epidemiol Infect. 2026 Oct 15;154:e130. doi: 10.1017/S0950268826102118 (PMC13749186; doi:10.1017/S0950268826102118)
Supplement: Supplementary file 1 [file S0950268826102118sup002.docx]

# Protocol

# Ethnicity in mathematical models of infectious respiratory diseases: a systematic review

## Investigators

Zoë Kumbaroff (corresponding and lead author), PhD candidate, Te Ngira Institute for Population Research, University of Waikato; Te Pūnaha Matatini Centre of Research Excellence for complex systems. Email: zmk1@students.waikato.ac.nz

Dr Jesse Whitehead, Te Ngira Institute for Population Research, University of Waikato; Te Pūnaha Matatini Centre of Research Excellence for complex systems.

Dr Dion O’Neale, Nicholson Consulting; Te Pūnaha Matatini Centre of Research Excellence for complex systems.

Dr Emily Harvey, Auckland City Council; Te Pūnaha Matatini Centre of Research Excellence for complex systems.

## Keywords

Health inequities, infectious diseases, mathematical modelling, epidemiology, public health, respiratory infections

## Background

Health inequities describe the unfair and avoidable differences in health outcomes experienced by some groups of people (World Health Organization, n.d.), for example, Indigenous, Black, and Hispanic ethnic groups. Since the coronavirus disease 2019 (COVID-19) pandemic, mathematical models have become commonly used tools in epidemiology for investigating infectious disease dynamics in human populations (Naidoo et al. 2024; Sweileh 2022). There is now a wealth of epidemiology modelling studies, particularly for evaluating COVID-19 related public health interventions. However, few models represent ethnic groups and report model outputs by ethnicity (Abuelezam et al. 2023; Richard and Lipsitch 2024; John-Baptiste et al. 2024). This is concerning, as total population analyses can hide what is occurring at the group level, thus hiding ethnic inequities. To date, there are no systematic reviews the investigate considerations of ethnicity in mathematical modelling. However, an understanding of how ethnicity can be appropriately captured and reported on in mathematical models is necessary for building further useful models that can be appropriately applied as tools for assisting in evaluating public health policy. An equity focus aligns with the principles of epidemiology – to understand the drivers and distribution of disease and thus should be at the forefront of mathematical epidemiological modelling.

This protocol describes the methods for systematic review that will be conducted to investigate the use of ethnicity in mathematic models for respiratory infection transmission in human populations – a topic which, to our knowledge has never been systematically summarized.

## Objectives

The objective of this narrative systematic review is to understand how ethnicity is considered in respiratory infectious disease mathematical modelling literature. Considerations include the representation of ethnicity in models as well as in the reporting of results and application to the real-world. The questions to be answered are:

1. How is ethnicity considered in mathematical models for respiratory infectious disease?
2. Where could bias arise and what steps are taken to mitigate potential biases?

## Methods/Design

This systematic review is being designed in line with The Preferred Reporting Items for Systematic reviews and Meta-Analyses (PRISMA) (Page et al. 2021).

We will search for mathematical modelling studies of infectious disease dynamics for pathogens that transmit from person to person through aerosols, for example, respiratory viruses such as influenza, SARS-CoV-2, measles, and tuberculosis.

#### Eligibility criteria

Characteristics and justification required of studies to be included in this review are:

- Mathematical model
- Contain an infectious disease transmission model (such as susceptible-infected-recovered (SIR) or extended version of this)
- Transmission model is applied to the human population in a particular geographic location or locations
- Infectious disease dynamics for a respiratory viral pathogen (transmitted through aerosols)
- Model results are reported for each included ethnic group
- Available in English

Exclusion criteria

- Model population restricted to vulnerable or high-risk group (e.g. isolated Indigenous groups)
- Non-communicable disease
- Infectious pathogens that are sexually/blood transmitted (HIV, STIs, HPV, hepatitis C), vector borne (e.g. malaria, Dengue fever), food/water borne.
- Non-human populations
- Publications with insufficient description of methods

Studies that restrict the model population to one ethnic group will not be included in the main review. Although this modelling method may have an equity focus by restricting analysis to a vulnerable population, it does not provide a comparator group for assessing inequities and is out of the scope of this review.

#### Information sources

Key words will be searched on Wiley, Cochrane, EBSCO host web, Scopus, and ProQuest electronic databases with no date limits. We limited the keyword search to abstract and filtered to returns that had published in English.

#### Search methods

Key word searches have been developed around three essential topics:

1. Mathematical modelling
2. Infectious diseases
3. Ethnicity

In addition, ‘NOT’ has been added to remove HIV and STI specific papers. These papers are not included in the review due to the vastly different infectious disease dynamics and contact patterns compared to respiratory pathogens.

The following key word search has been developed for the Wiley electronic database:

| **Concept** | **Key words** |
| --- | --- |
| Mathematical modelling | ("agent based" OR "individual based" OR "network" OR "compartmental" OR "meta population" OR "SEIR" OR "SIR" OR "SEIRS" OR "SEIRD" OR "ODE" OR "SDE" OR "branching process" OR "metapopulation" OR "population" OR "dynamical systems" OR "mathematical" OR simulat*) AND |
| Model | MODEL* AND |
| Infectious diseases | ("SARS-CoV-2" OR measles OR meningococcal OR pneumococcal OR rhinovirus OR metapneumovirus OR hmpv OR parainfluenza OR "common cold" OR influenza OR rsv OR "COVID" OR coronavirus OR infect* OR tuberculos* OR "respiratory illness" OR "Respiratory syncytial virus") AND |
| Ethnicity | (ethnic* OR race OR racial OR indig* OR "First nation" OR "native american" OR "African American" OR aboriginal* OR māori OR "Pasifika" OR "Pacific Island" OR "Pacific people" OR hispanic OR latin* OR hawaiian OR inuit OR "alaska native" OR minority OR roma* OR refugee OR marginali*) |
| Irrelevant pathogens | NOT HIV NOT STI NOT STD NOT HPV |

The following key word search has been developed for the Cochrane database:

| **Concept** | **Keywords** |
| --- | --- |
| Mathematical modelling | ("agent based" OR "individual based" OR "network" OR "compartmental" OR "meta population" OR "SEIR" OR "SIR" OR "SEIRS" OR "SEIRD" OR "ODE" OR "SDE" OR "branching process" OR "metapopulation" OR "population" OR "dynamical systems" OR "mathematical" OR simulat*) AND |
| Model | MODEL* AND |
| Infectious diseases | ("SARS-CoV-2" OR measles OR meningococcal OR pneumococcal OR rhinovirus OR metapneumovirus OR hmpv OR parainfluenza OR "common cold" OR influenza OR rsv OR "COVID" OR coronavirus OR infect* OR tuberculos* OR "respiratory illness" OR "Respiratory syncytial virus") AND |
| Ethnicity | (ethnic* OR race OR racial OR indig* OR "First nation" OR "native american" OR "African American" OR aboriginal* OR māori OR "Pasifika" OR "Pacific Island" OR "Pacific people" OR hispanic OR latin* OR hawaiian OR inuit OR "alaska native" OR minority OR roma* OR refugee OR marginali*) |
| Irrelevant pathogens | NOT HIV NOT STI NOT STD NOT HPV |

The following key word search has been developed for the EBSCO host web electronic database:

| **Concept** | **Key words** |
| --- | --- |
| Mathematical modelling | ( "agent based model*" OR "individual based model*" OR "network model*" OR "compartmental model*" OR "metapopulation model*" OR "SEIR model*" OR "SIR model*" OR "SEIRS model*" OR "SEIRD model" OR "ODE model*" OR "SDE model*" OR "branching process model*" OR "meta-population model*" OR "population model*" OR "dynamical systems model*" OR "mathematical model*" OR simulat* ) AND |
| Infectious diseases | ( "SARS-CoV-2" OR measles OR meningococcal OR pneumococcal OR rhinovirus OR metapneumovirus OR hmpv OR parainfluenza OR "common cold" OR influenza OR rsv OR "COVID*" OR coronavirus OR "infect*" OR tuberculosis OR "respiratory illness" OR "Respiratory syncytial virus" ) AND |
| Ethnicity | ( ethnic* OR race OR racial OR indig* OR "First Nation*" OR "Native American" OR "African American" OR aboriginal* OR māori OR "Pasifika" OR "Pacific Island*" OR "Pacific people*" OR hispanic OR latin* OR hawaiian OR inuit OR "Alaska native" OR minority OR roma* OR refugee OR marginali* ) |
| Irrelevant pathogens | AND NOT hiv OR sti OR std OR hpv |

The following key word search has been developed for the Scopus electronic database:

| **Concept** | **Key words** |
| --- | --- |
| Mathematical modelling | TITLE-ABS-KEY(("agent based model*" OR "individual based model*" OR "network model*" OR "compartmental model*" OR "metapopulation model*" OR "SEIR model*" OR "SIR model*" OR "SEIRS model*" OR "SEIRD model" OR "ODE model*" OR "SDE model*" OR "branching process model*" OR "meta population model*" OR "population model*" OR "dynamical systems model*" OR "mathematical model*" OR simulat* ) |
| Infectious diseases | ("SARS-CoV-2" OR measles OR meningococcal OR pneumococcal OR rhinovirus OR metapneumovirus OR hmpv OR parainfluenza OR "common cold" OR influenza OR rsv OR "COVID*" OR coronavirus OR "infect*" OR tuberculosis OR "respiratory illness" OR "Respiratory syncytial virus" ) AND |
| Ethnicity | (ethnic* OR race OR racial OR indig* OR "First Nation*" OR "Native American" OR "African American" OR aboriginal* OR māori OR "Pasifika" OR "Pacific Island*" OR "Pacific people*" OR hispanic OR latin* OR hawaiian OR inuit OR "Alaska native" OR minority OR roma* OR refugee OR marginali* ) AND |
| Irrelevant pathogens | AND NOT (hiv OR sti OR std OR hpv)) |

The following key word search has been developed for the Proquest electronic database:

| **Concept** | **Key words** |
| --- | --- |
| Mathematical modelling | ( "agent based model*" OR "individual based model*" OR "network model*" OR "compartmental model*" OR "metapopulation model*" OR "SEIR model*" OR "SIR model*" OR "SEIRS model*" OR "SEIRD model" OR "ODE model*" OR "SDE model*" OR "branching process model*" OR "meta population model*" OR "population model*" OR "dynamical systems model*" OR "mathematical model*" OR simulat* ) AND |
| Infectious diseases | ( "SARS-CoV-2" OR measles OR meningococcal OR pneumococcal OR rhinovirus OR metapneumovirus OR hmpv OR parainfluenza OR "common cold" OR influenza OR rsv OR COVID* OR coronavirus OR infect* OR tuberculosis OR "respiratory illness" OR "Respiratory syncytial virus" ) AND |
| Ethnicity | (ethnic* OR race OR racial OR indig* OR "First Nation*" OR "Native American" OR "African American" OR aboriginal* OR māori OR "Pasifika" OR "Pacific Island*" OR "Pacific people*" OR hispanic OR latin* OR hawaiian OR inuit OR "Alaska native" OR minority OR roma* OR refugee OR marginali* ) |
| Irrelevant pathogens | AND NOT hiv OR sti OR std OR hpv |

Citation lists from the included literature will then be scanned to identify any further relevant studies. Web searches will also be conducted to find any other relevant material.

#### Study records

All references will be managed using Endnote, and literature screening will be conducted using a combination of Endnote and Excel.

References will be imported to Endnote and deduplicated. References will then be exported to Excel. All references will be scanned by title for relevance. Full text for the relevant references will then be screened against the inclusion criteria to determine the final set of studies for inclusion. Title scanning and full text screening will be conducted by one researcher and checked by a second researcher.

Data related to the outcomes of interest will be extracted from the final set of studies and summarized in an excel spreadsheet.

#### Data synthesis

A qualitative comparison of studies will discuss the key themes identified throughout literature. The qualitative analysis will include:

- PRISMA inclusion/exclusion flowchart
- A table of data extraction results for the outcomes of interest
- Discussion of results.

The outcomes of interest are:

- Setting
  - Geographical location of population
  - Demographic groups considered including synthetic or hypothetical populations
- Purpose of model
- Methods and features
  - Model framework
  - Pathogen
  - Population demographic groups
  - Data sources for model population, contact patterns, disease parameters, model calibration
  - How ethnicity is used in the model
- Findings by ethnicity
- How ethnic inequities are discussed
- Limitations
- Biases
- Reproducibility

#### Risk of bias in individual studies

Studies will be assessed based on their likelihood of bias arising from data sources, modelling methods, and how results are reported. Mathematical model assessment frameworks have been summarised by (Egger et al. 2018) which will guide the assessment of bias in our systematic review. We will focus on whether authors discuss data biases and model limitations, identify funders and any conflicts of interest, and attempt to make models reproducible through publishing code and data.

## References

Abuelezam, Nadia N., Isaacson Michel, Brandon Dl Marshall, and Sandro Galea. 2023. “Accounting for Historical Injustices in Mathematical Models of Infectious Disease Transmission: An Analytic Overview.” *Epidemics* 43 (June):100679. https://doi.org/10.1016/j.epidem.2023.100679.

Egger, Matthias, Leigh Johnson, Christian Althaus, Anna Schöni, Georgia Salanti, Nicola Low, and Susan L. Norris. 2018. “Developing WHO Guidelines: Time to Formally Include Evidence from Mathematical Modelling Studies.” *F1000Research* 6 (February):1584. https://doi.org/10.12688/f1000research.12367.2.

John-Baptiste, Ava A., Marc Moulin, Zhe Li, Darren Hamilton, Gabrielle Crichlow, Daniel Eisenkraft Klein, Feben W. Alemu, et al. 2024. “Do COVID-19 Infectious Disease Models Incorporate the Social Determinants of Health? A Systematic Review.” *Public Health Reviews* 45 (October):1607057. https://doi.org/10.3389/phrs.2024.1607057.

Naidoo, Megan, Whitney Shephard, Innocensia Kambewe, Nokuthula Mtshali, Sky Cope, Felipe Alves Rubio, and Davide Rasella. 2024. “Incorporating Social Vulnerability in Infectious Disease Mathematical Modelling: A Scoping Review.” *BMC Medicine* 22 (1): 125. https://doi.org/10.1186/s12916-024-03333-y.

Page, Matthew J, David Moher, Patrick M Bossuyt, Isabelle Boutron, Tammy C Hoffmann, Cynthia D Mulrow, Larissa Shamseer, et al. 2021. “PRISMA 2020 Explanation and Elaboration: Updated Guidance and Exemplars for Reporting Systematic Reviews.” *BMJ*, March, n160. https://doi.org/10.1136/bmj.n160.

Richard, Danielle M, and Marc Lipsitch. 2024. “What’s next: Using Infectious Disease Mathematical Modelling to Address Health Disparities.” *International Journal of Epidemiology* 53 (1): dyad180. https://doi.org/10.1093/ije/dyad180.

Sweileh, Waleed M. 2022. “Global Research Activity on Mathematical Modeling of Transmission and Control of 23 Selected Infectious Disease Outbreak.” *Globalization and Health* 18 (1): 4. https://doi.org/10.1186/s12992-022-00803-x.

World Health Organization. n.d. “Health Equity.” Accessed September 3, 2024. https://www.who.int/health-topics/health-equity.
